# Clomiphene Citrate Treatment Cycle Outcomes of Polycystic Ovary Syndrome Patients Based on Basal High Sensitive C-Reactive Protein Levels: A Cross-Sectional Study

**DOI:** 10.22074/ijfs.2016.4849

**Published:** 2016-11-01

**Authors:** Serkan Kahyaoglu, Omer Hamid Yumuşak, Sebnem Ozyer, Meryem Kuru Pekcan, Merve Erel, Mahmut Nedim Cicek, Salim Erkaya, Yasemin Tasci

**Affiliations:** Department of Obstetrics and Gynecology, Zekai Tahir Burak Women’s Health Education and Research Hospital, Ankara, Turkey

**Keywords:** Polycystic Ovary Syndrome, Ovulation Induction, Clomiphene, CReactive Protein

## Abstract

**Background:**

Polycystic ovary syndrome (PCOS) is highly associated with an ovulatory infertility, features of the metabolic syndrome, including obesity, insulin resistance and dyslipidemia. Serum concentrations of high sensitive C-reactive protein (hs-CRP) were significantly higher in obese than in non-obese PCOS patients at baseline, suggesting a relationship between elevated hs-CRP levels and obesity. The aim of this study was to evaluate whether cycle day 3 hs-CRP levels before clomiphene citrate (CC) treatment would predict cycle outcomes in women with PCOS.

**Materials and Methods:**

This cross-sectional study was conducted among 84 infertile women with PCOS who were treated with CC at Zekai Tahir Burak Women’s Health Education and Research Hospital, Ankara, Turkey, between January 2014 and January 2015. Based on the exclusion criteria, cycle outcomes of remaining 66 infertile women with PCOS treated with CC were analyzed. The hs-CRP levels and insulin resistance indexes were evaluated on day 3 of the CC treatment cycle. The primary outcome measures were number of preovulatory follicles measuring≥17 mm and pregnancy rates.

**Results:**

The mean ± SD age of the patients was 24.0 ± 3.8 years (range 18-36). The mean ± SD body mass index (BMI) of the patients was 25.7 ± 4.9 (range 17-43). Fifty patients developed dominant follicle (75%) and 5 patients established clinical pregnancy during the study (clinical pregnancy rate: 7%). The mean ± SD baseline hs-CRP, fasting insulin and Homeostasis Model Assessment-Insulin Resistance (HOMA-IR) values of the patients with and without dominant follicle generation during treatment cycle were 6.42 ± 7.05 and 4.41 ± 2.95 (P=0.27), 11.61 ± 6.94 and 10.95 ± 5.65 (P=0.73), 2.68 ± 1.79 and 2.41 ± 1.30 (P=0.58), respectively. The mean ± SD baseline hs-CRP, fasting insulin and HOMA-IR values of the patients with and without clinical pregnancy establishment following treatment cycle were 6.30 ± 2.56 and 5.90 ± 6.57 (P=0.89), 11.60 ± 7.54 and 11.44 ± 6.61 (P=0.95), 2.42 ± 1.51 and 2.63 ± 1.70 (P=0.79), respectively.

**Conclusion:**

In this study, we did not observe a predictive value of cycle day 3 hs-CRP levels on preovulatory follicle development and pregnancy rates among infertile PCOS patients treated with CC. Also, no relationship between HOMA-IR values and dominant follicle generation or clinical pregnancy establishment was demonstrated in our study, confirming the previous studies emphasizing the neutral effect of metformin utilization before and/or during ovulation induction to pregnancy rates.

## Introduction

Polycystic ovary syndrome (PCOS) is the most common endocrine disorder with a 5-10% prevalence among women of reproductive age (1,3). PCOS should be diagnosed with the presence of two of three criteria (oligo-ovulation/anovulation, hyperandrogenism, and polycystic ovarian morphology) on ultrasonography after exclusion of other endocrine disorders as determined at the 2003 Rotterdam consensus meeting (4). Hyperandrogenism has been established as a mandatory diagnostic criteria for PCOS in National Institute of Health (NIH) and Androgen Excess Society criteria unlike Rotterdam consensus. PCOS is strongly related to irregular menstrual cycles, oligo-anovulation, and infertility accompanied by metabolic disorders like obesity, insulin resistance, gestational diabetes mellitus, diabetes, and cardiovascular disease, which makes this relatively prevalent syndrome as a public health issue (5,7). 

Chronic low-grade inflammation is involved in the pathogenesis of obesity-related syndromes like PCOS, which is also a proinflammatory state with an association between inflammation at the molecular level and insulin resistance (8,11). It still remains unclear that whether elevations of inflammatory markers like tumor necrosis factoralpha (TNF-α), interleukin-6 (IL-6) and high sensitive C-reactive protein (hs-CRP) are related to PCOS or are a function of obesity, abdominal adiposity, or both. Serum concentrations of hsCRP were significantly higher in obese than in non-obese PCOS patients at baseline, suggesting a relationship between elevated hs-CRP levels and obesity (12). Clinical reflections of increased serum levels of hs-CRP among obese and nonobese PCOS patients are not clear and worth to investigate for determining a predictive marker for future prognosis of interventions in management of PCOS. Increased hs-CRP levels as a reflection of activated systemic inflammatory response either due to obesity or PCOS disease can be considered as increased metabolic activity of the disease. This increased inflammatory activity can theoretically affect folliculogenesis, and ovulation as the anovulation is the main defect of patients with PCOS, resulting in infertility. The aim of this study was to evaluate whether cycle day 3 hs-CRP levels before commencing clomiphene citrate (CC) treatment would predict ovulation induction cycle outcomes in women with PCOS. 

## Materials and Methods

This cross-sectional study was conducted at the infertility clinic of Zekai Tahir Burak Women’s Health Education and Research Hospital, Ankara, Turkey, between January 2014 and January 2015. PCOS patients with tubal factor infertility, male infertility, endometriosis, and systemic disorders like overt diabetes mellitus, cardiac pathologies and thyroid diseases were excluded. The cycle outcomes of 84 infertile women with PCOS who were treated with CC+coitus or CC+intrauterine insemination (IUI) were evaluated. Eighteen patients were excluded due to use of metformin and absence of completing the treatment cycle. Remaining 66 patients’ ovulation induction cycle data were assessed. Of 66 patients, 6 patients’ pregnancy outcomes were not detected due to loss from follow-up. The hs-CRP levels of the patients were measured on cycle day 3 of the CC treatment cycle. The serum samples for hs-CRP levels were drawn on the day 3 of the menstrual cycle because ovulation induction stimulates an inflammatory response. hs-CRP was measured with a high sensitivity immunoturbidimetric assay (Roche Diagnostics, USA) using an automated clinical chemistry analyzer. The hs-CRP assay coefficients of variation were 2.7% at 0.12 mg/L, 3.45% at 0.41 mg/L, and 5.7% at 0.03 mg/L. The assay has a detection limit of 0.03 mg/L and a calibration range up to 300 mg/L. Normal range reference value of hs-CRP for adults was accepted as <5 mg/dL. 

Insulin resistance situation of the patients was evaluated using the Homeostasis Model Assessment-Insulin Resistance (HOMA-IR) within the 2 months preceding the treatment cycle. HOMA-IR values greater than 2.5 were thought to be related to insulin resistance (13). Then, patients were given 50-100 mg CC for five days starting from day three of their menstrual cycles. A transvaginal ultrasonography was performed at day 12 and every other day until a follicle ≥ 17 mm was detected. Timely coitus or IUI procedure regarding the preference of the patients and clinicians was recommended to the patients 36 hours after triggering the ovulation with 10.000 IU human chorionic gonadotropin (hCG) intramuscular injection. Patients underwent a serum pregnancy test on the 14^th^day following triggering of ovulation. Patients with positive pregnancy tests were called for evaluation of clinical pregnancy with ultrasonography at 5 weeks after the pregnancy test. Clinical pregnancy was defined as fetal heart beat detection on transvaginal ultrasonography at 7 weeks of gestation. The primary outcome measures were number of preovulatory follicles measuring≥17 mm and pregnancy rates. 

### Ethical considerations

An informed consent was obtained from all of participants, and the Institutional Review Board of Zekai Tahir Burak Women's Health Education and Research Hospital approved this project. 

### Statistical analysis

Statistical analysis was performed using Statistical Package for the Social Sciences (SPSS, SPSS Inc., USA) version 22.0. Normal distribution of data was evaluated using Kolmogorov-Smirnov test. The continuous variables were presented by mean ± SD and compared using the independent sample t test. The nonparametric variables without normal distribution were tested using Mann Whitney U test. Correlation analysis was performed using Pearson correlation test. The comparison of categorical values was made using Fisher’s exact test or Chi-square test. Receiver operating characteristic (ROC) curve was used to compare the diagnostic performance of the diagnostic tests. P<0.05 were considered statistically significant. 

## Results

The mean ± SD age of the patients was 24.0 ± 3.8 years (range 18-36) with a mean duration of infertility of 3.0 ± 1.6 years (range 1-9). The mean ± SD body mass index (BMI) of the patients was 25.7 ± 4.9 (range 17-43). The mean ± SD cycle day 3 hsCRP level of the patients was 5.93 ± 6.35 and the mean ± SD HOMA-IR value of the patients was 2.61 ± 1.68. Fifty patients developed dominant follicle (75%) and 5 patients established clinical pregnancy during the study (clinical pregnancy rate: 7%). All patients (5 of 5) with clinical pregnancy establishment and 33 of 55 patients (60%) without clinical pregnancy establishment underwent an IUI procedure despite the fact that male infertility was an exclusion criteria of the study (P=0.64). The baseline hs-CRP levels and HOMA-IR values of the patients were not correlated (r=0.02 using Pearson correlation, P=0.87). A significantly positive correlation was seen between baseline hs-CRP levels and BMI values of the patients (r=0.54 using Pearson correlation; P<0.001). CC treatment cycle days 3 and 5 did not influence the dominant follicle development (P=0.37) and clinical pregnancy achievement (P=0.47), respectively. Dominant follicle development following CC treatment was not related to baseline insulin resistance (P=0.64). The mean ± SD baseline hs-CRP, fasting insulin and HOMA-IR values of the patients with and without dominant follicle generation during treatment cycle were 6.42 ± 7.05 and 4.41 ± 2.95 (P=0.27), 11.61 ± 6.94 and 10.95 ± 5.65 (P=0.73), 2.68 ± 1.79 and 2.41 ± 1.30 (P=0.58), respectively. 

The ovulation induction cycle outcomes of the study group according to establishment of clinical pregnancy following treatment cycle are demonstrated in Table 1. Patients’ ovulation induction cycle outcomes based on BMI classification are demonstrated in Table 2. 

**Table 1:** Cycle outcomes of patients with and without achieving clinical pregnancy with ovulation induction by administration of 5 days of oral CC treatment


Parameter	Clinical pregnancy (+)(n=5)	Clinical pregnancy (-)(n=55)	P value

Age (Y)	22.4 ± 2.5	24.6 ± 3.9	0.32^*^
BMI (ratio)	27.0 ± 5.0	25.1 ± 4.4	0.37^*^
Infertility duration (Y)	3.0 ± 1.2	3.0 ± 1.5	0.54^*^
Day 3
FSH (mIU/mL)	5.8 ± 2.3	6.2 ± 1.2	0.70^**^
LH (mIU/mL)	7.1 ± 4.8	8.8 ± 6.4	0.43^**^
E_2_ (pg/mL)	55 ± 35	45 ± 16	0.90^**^
Cycle day of CC commencement n (%)
Day 3	5 (100%)	47 (85.5%)	0.47^***^
Day 5	0	8 (14.5)	
Day 3 CRP level (mg/dL)	4.58 ± 2.25	5.94 ± 6.91	0.75
Fasting glucose level (mg/dL)	91.5 ± 8.8	89.9 ± 9.9	0.72^**^
2-hour postprandial glucose level (mg/dL)	106.9 ± 19.7	105.8 ± 21.8	0.91^**^
Fasting insulin (mIU/L)	10.2 ± 3.6	11.3 ± 6.6	0.90^*^
HOMA-IR (ratio)	2.25 ± 0.65	2.59 ± 1.72	0.78^*^
>14 mm follicle number (n)	0.8 ± 0.4	1.2 ± 1.2	0.51^*^
Day 12 periovulatuar E_2_ level (pg/mL)	124 ± 62	550 ± 687	0.17^*^
hCG triggering day of cycle	17.01.4	13.52.9	0.015^*^
hCG day endometrial thickness (mm)	7.0 ± 1.8	7.2 ± 1.8	0.73^**^


Data are presented as mean ± SD, ^*^; Mann Whitney U test, ^**^; Independent samples t test, ^***^; Fisher’s exact test, CC; Clomiphene citrate, BMI; Body mass index, CRP; C-reactive protein, FSH; Follicle stimulating hormone, LH; Luteinizing hormone, E_2_ ; Estardiol, hCG; Human chorionic gonadotropin, and HOMA-IR; Homeostasis model assessment-insulin resistance.

**Table 2:** Patients’ ovulation induction cycle outcomes based on BMI classification


BMI (kg/height^2^)	Cycle day 3hs-CRP	Fasting glucose level	2-hour postprandial glucose level following 75 g oral glucose tolerance test	HOMA-IR	Number of follicle>14 mm following ovulation induction	Dominant follicle achievement n(%)	Clinical pregnancy achievement n(%)

<30 (n=52)Mean ± SD	5.68 ± 6.88	89.4 ± 10.0	105.4 ± 21.4	2.46 ± 1.75	1.24 ± 1.33	29 (55%)	2 (3.8%)
≥30 (n=8)Mean ± SD	6.79 ± 5.08	94.0 ± 7.8	108.4 ± 22.6	3.23 ± 0.62	1.13 ± 0.35	8 (100%)	3 (37.5%)
P value	0.24^*^	0.10^*^	0.62^*^	0.01^*^	0.76^*^	0.09^**^	0.01^**^
Total (n=60)Mean ± SD, n (%)	5.83 ± 6.64	90.0 ± 9.8	105.9 ± 21.4	2.56 ± 1.66	1.23 ± 1.23	37 (61%)	5 (8%)


^*^; Mann Whitney U test, ^**^; Fisher’s exact test, BMI; Body mass index, and HOMA-IR; Homeostatic model of assessment-insulin resistance.

**Fig.1:**
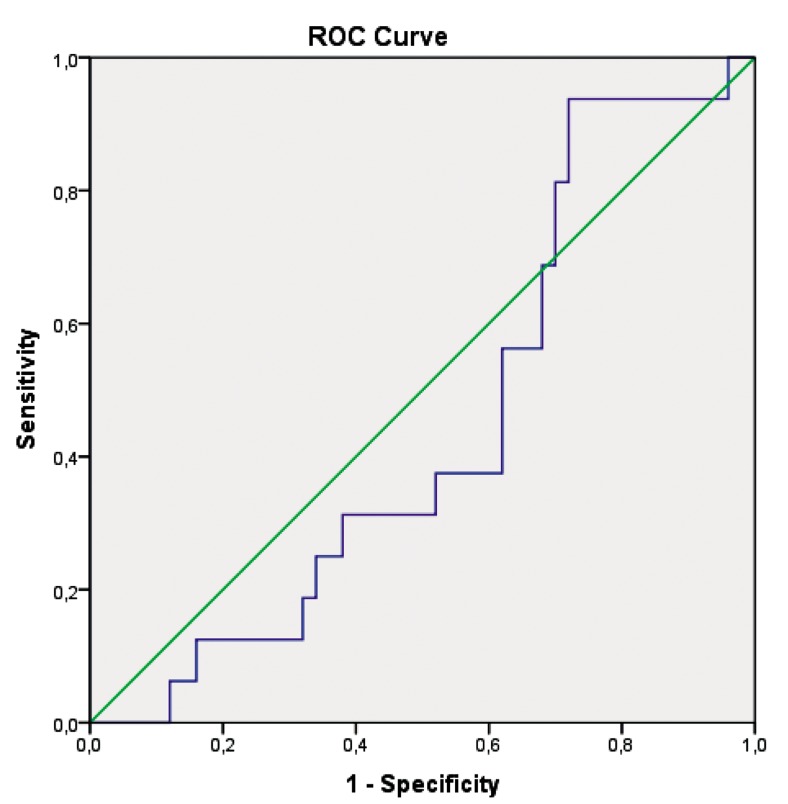
ROC analysis of cycle day 3 hs-CRP levels and dominant follicle establishment following 5 days CC treatment for ovulation induction (AUC=0.44, 95% CI=0.29-0.59, P=0.52). ROC; Receiver operating curve, hs-CRP; High sensitive C-reactive protein, CC; Clomiphene citrate, AUC; Area under the curve, and CI; Confidence interval.

Our findings showed that cycle day 3 hs-CRP levels, fasting serum glucose levels and 2-hour postprandial serum glucose levels were found to be higher among patients with BMI values ≥30 than patients with BMI values <30, indicating there were statistically insignificant differences in this regard. HOMA-IR value and clinical pregnancy rate were significantly higher in patients with BMI values ≥30 than patients with BMI values <30. Cycle day 3 hs-CRP levels showed no predictive value for dominant follicle establishment following 5 days CC treatment, [area under the curve (AUC)=0.44, 95% confidence interval (CI)=0.29-0.59, P=0.52] (Fig .1).

**Fig.2:**
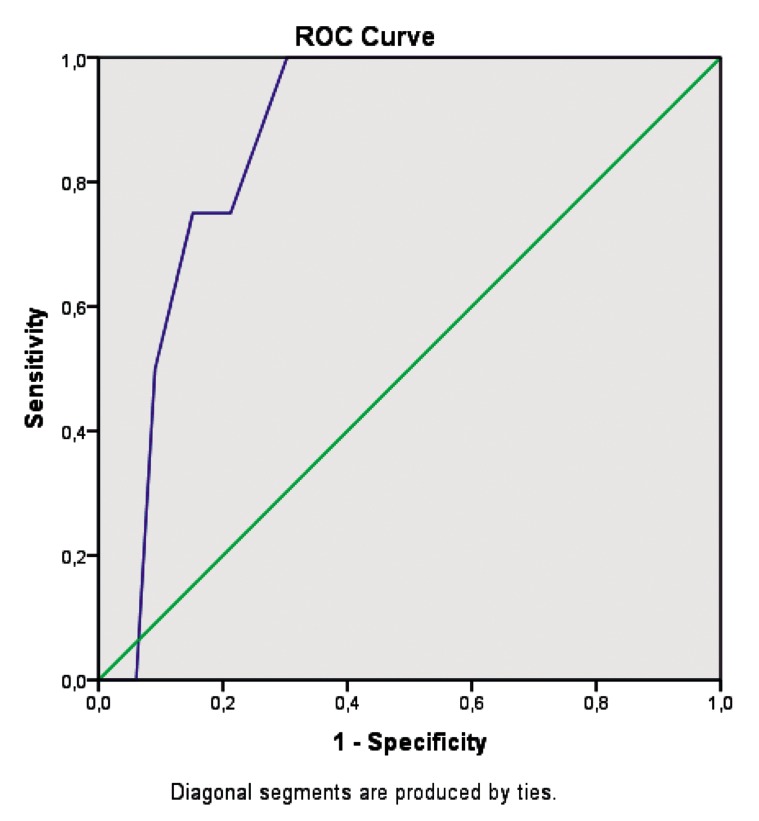
ROC analysis of ovulation triggering day by hCG and clinical pregnancy achievement (AUC=0.86, 95% CI=0.74-0.99, P=0.018). Cut-off value for predicting clinical pregnancy establishment by ovulation triggering day is 16.5 days with sensitivity and specificity of 75 and 85%, respectively. ROC; Receiver operating curve, hCG; human chorionic gonadotropin, AUC; Area under the curve, and CI; Confidence interval.

Later ovulation triggering cycle day by hCG utilization significantly predicted successful pregnancy outcome (AUC=0.86, 95% CI=0.74-0.99, P=0.018) (Fig .2). 

Later ovulation triggering cycle day by hCG utilization significantly predicted successful pregnancy outcome (AUC=0.86, 95% CI=0.74-0.99, P=0.018) (Fig .2).

Cut-off value for predicting clinical pregnancy establishment by ovulation triggering day is 16.5 days with sensitivity and specificity of 75 and 85%, respectively. When 2.5 cut-off level was considered for increased HOMA-IR ratio reflecting insulin resistance, high HOMA-IR ratio was not correlated with dominant follicle development (P=0.64) and clinical pregnancy establishment (P=0.65). No statistically significant relationship was determined between BMI and dominant follicle generation or clinical pregnancy establishment. 

## Discussion

PCOS is a multisystemic disorder, which includes short and long-term complications for the affected women. Exact pathophysiological mechanism and etiological factors have not been yet defined. Serum levels of inflammatory markers like hs-CRP are elevated in PCOS which demonstrates chronic low-grade inflammation state of the disorder. Serum hs-CRP level is positively correlated with adipose tissue content and BMI ratio of the body (14). In our study, we also demonstrated a significantly positive relationship between hs-CRP and high BMI levels. The short and long-term clinical effects of high hs-CRP level caused by high BMI level cannot be predicted. Success rate of ovulation induction cycles among PCOS patients are needed to be evaluated with objective laboratory or clinical markers. We hypothesized that high cycle day 3 serum level of hs-CRP would deteriorate folliculogenesis that resulted in unfavorable cycle outcomes reflected by absence of dominant follicle and implantation failure. However, we could not detect any significant relationship between serum hs-CRP levels and cycle outcomes. Later artificial ovulation triggering day with hCG was significantly associated with clinical pregnancy achievement in our study. We established 16.5 days of artificial ovulation triggering day from the beginning of the menstrual cycle as a cut-off level for successful cycle outcomes induced by CC in PCOS patients. This can be explained by relatively late maturation of oocytes in CC cycles unlike gonadotropin cycles (15). 

In this study, we did not observe a predictive value of cycle day 3 hs-CRP levels on preovulatory follicle development and pregnancy rates among infertile PCOS patients treated with CC. Although weight reduction to improve folliculogenesis in PCOS patients is recommended as a first line management strategy, the benefits of getting rid of obesity seems to be achieved by other pathophysiological events rather than lowering serum hs-CRP levels. HOMA-IR levels and clinical pregnancy rates of the patients with BMI values ≥30 were found to be significantly higher than patients with BMI values <30, which demonstrates that HOMA-IR levels did not adversely affect pregnancy achievement among obese PCOS patients. Cycle day 3 hs-CRP levels and dominant follicle achievement rates of patients with BMI values ≥30 were found to be insignificantly higher than patients with BMI values <30, which demonstrates that cycle day 3 hs-CRP levels, as a reflection of systemic inflammatory response, did not adversely or positively affect folliculogenesis and clinical pregnancy establishment. Agacayak et al. (16) have evaluated the levels of inflammatory markers and neopterin in obese and non-obese patients with PCOS using 2 separate control groups with matching BMI. No statistically significant difference was found between obese and non-obese patients with PCOS and control subjects in neopterin, IL-6, TNF-α, and neutrophil/lymphocyte ratio levels unlike CRP levels which were significantly higher in obese patients with PCOS compared to obese control subjects, demonstrating the facilitatory effect on serum CRP levels of PCOS patients by disease itself rather than obesity. Aziz et al. (17) have also demonstrated the relationship between obesity, insulin resistance and CRP with plasma endogen thrombin generation, which reflects cardiovascular disease progression among patients with PCOS. 

Orvieto et al. (18) have investigated serum and follicular fluid CRP levels in patients undergoing controlled ovarian hyperstimulation (COH) for *in vitro* fertilization (IVF)-embryo transfer cycle and their possible correlation to COH variables. In this study, a significant increase in serum CRP levels during COH, especially after hCG administration, has been detected during COH procedure which demonstrates the stimulatory effect of COH on a state of systemic inflammation. They have found that no significant correlations exist between serum and follicular fluid CRP, or between serum CRPto-BMI ratio and serum sex steroid levels or IVF treatment variables. In another study performed by the same group, a significant increase in serum ovarian androgen levels during gonadotropin treatment has been detected in patients undergoing COH for IVF. A significant increase in the levels of both serum CRP and ovarian androgens (testosterone, androstenedione) has also been found after hCG administration, which demonstrates that ovarian androgen levels increase in correlation with the degree of inflammation, as reflected by CRP levels (19). The results of this study supported our objective regarding the association between increased systemic inflammatory response accompanied by increased ovarian androgen levels and defective folliculogenesis among patients with PCOS. 

Secondly, no relationship between HOMA-IR values and dominant follicle generation or clinical pregnancy establishment were demonstrated in our study, confirming the previous studies emphasizing the absence of any beneficial effect of metformin utilization on pregnancy rates before and/or during ovulation induction (20). The number of clinical pregnancies was so low to make a conclusive statement for the relationship between baseline hs-CRP levels and pregnancy rate. 

## Conclusion

Both basal serum hs-CRP levels and HOMA-IR ratios showed no predictive value for development of dominant follicles and/or achievement of clinical pregnancy among infertile women diagnosed with PCOS. Further studies with larger number of patients for evaluating the predictive value of hsCRP on cycle outcomes of infertile PCOS patients are needed. 
